# Elongation factor 1A1 inhibition elicits changes in lipid droplet size, the bulk transcriptome, and cell type-associated gene expression in MASLD mouse liver

**DOI:** 10.1152/ajpgi.00276.2023

**Published:** 2024-08-13

**Authors:** Rachel B. Wilson, Yun Jin Chen, Richard Zhang, Siddhant Maini, Tallulah S. Andrews, Rennian Wang, Nica M. Borradaile

**Affiliations:** ^1^Department of Physiology and Pharmacology, Schulich School of Medicine and Dentistry, Western University, London, Ontario, Canada; ^2^Children’s Health Research Institute, Lawson Health Research Institute, London, Ontario, Canada; ^3^Department of Biochemistry, Schulich School of Medicine and Dentistry, Western University, London, Ontario, Canada

**Keywords:** lipid droplet, liver biology, MASLD, EEF1A1

## Abstract

Eukaryotic elongation factor 1A1 (EEF1A1), originally identified for its role in protein synthesis, has additional functions in diverse cellular processes. Of note, we previously discovered a role for EEF1A1 in hepatocyte lipotoxicity. We also demonstrated that a 2-wk intervention with the EEF1A1 inhibitor didemnin B (DB) (50 µg/kg) decreased liver steatosis in a mouse model of obesity and metabolic dysfunction-associated steatotic liver disease (MASLD) [129S6/SvEvTac mice fed Western diet (42% fat) for 26 wk]. Here, we further characterized the hepatic changes occurring in these mice by assessing lipid droplet (LD) size, bulk differential expression, and cell type-associated alterations in gene expression. Consistent with the previously demonstrated decrease in hepatic steatosis, we observed decreased median LD size in response to DB. Bulk RNA sequencing (RNA-Seq) followed by gene set enrichment analysis revealed alterations in pathways related to energy metabolism and proteostasis in DB-treated mouse livers. Deconvolution of bulk data identified decreased cell type association scores for cholangiocytes, mononuclear phagocytes, and mesenchymal cells in response to DB. Overrepresentation analyses of bulk data using cell type marker gene sets further identified hepatocytes and cholangiocytes as the primary contributors to bulk differential expression in response to DB. Thus, we show that chemical inhibition of EEF1A1 decreases hepatic LD size and decreases gene expression signatures associated with several liver cell types implicated in MASLD progression. Furthermore, changes in hepatic gene expression were primarily attributable to hepatocytes and cholangiocytes. This work demonstrates that EEF1A1 inhibition may be a viable strategy to target aspects of liver biology implicated in MASLD progression.

**NEW & NOTEWORTHY** Chemical inhibition of EEF1A1 decreases hepatic lipid droplet size and decreases gene expression signatures associated with liver cell types that contribute to MASLD progression. Furthermore, changes in hepatic gene expression are primarily attributable to hepatocytes and cholangiocytes. This work highlights the therapeutic potential of targeting EEF1A1 in the setting of MASLD, and the utility of RNA-Seq deconvolution to reveal valuable information about tissue cell type composition and cell type-associated gene expression from bulk RNA-Seq data.

## Graphical abstract

**Figure:**
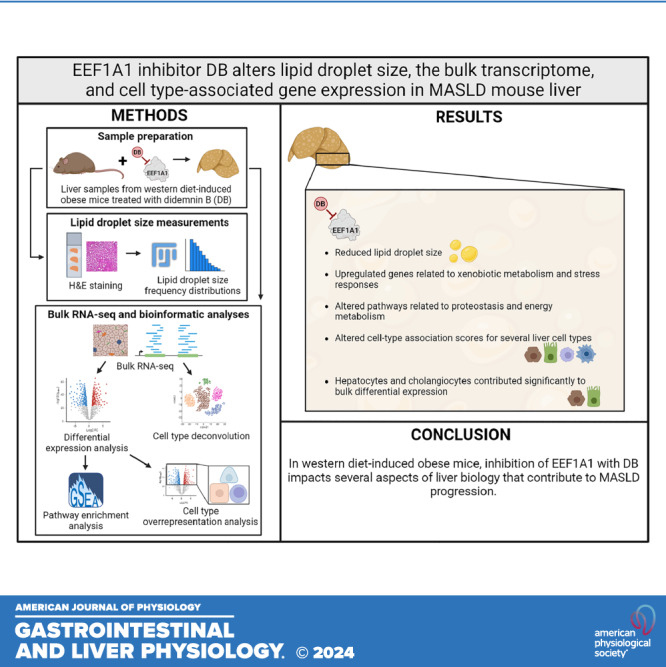

## INTRODUCTION

Metabolic dysfunction-associated steatotic liver disease (MASLD), formerly known as nonalcoholic fatty liver disease (NAFLD) (1), is the most common chronic liver disease and the leading cause of liver-related morbidity and mortality worldwide, with an estimated global prevalence of 32% (2). MASLD comprises a spectrum of diseases, the defining feature of which is the presence of lipid droplets (LDs) in at least 5% of hepatocytes, termed steatosis (3). Steatosis can be macrovesicular (macrosteatosis) or microvesicular (microsteatosis) in nature. Macrosteatosis presents either as one large LD or several smaller LDs that displace cytoplasmic contents and the nucleus to the cell periphery, whereas microsteatosis is characterized by an abundance of tiny LDs within the hepatocyte cytoplasm, which creates a foamy appearance (4). Microsteatosis has been associated with more advanced histological disease in MASLD (5). However, other studies suggest that macrosteatosis may be more detrimental than microsteatosis (6–9), and large LDs have been shown to exert intracellular mechanical stress, deform nuclei (10, 11), and impair hepatocyte function (11, 12). Thus, there are implications of LD size for MASLD severity and hepatocyte function, although the exact relationships are unclear.

Of patients with MASLD, 20%–25% will progress to metabolic dysfunction-associated steatohepatitis (MASH) (13), characterized by hepatocellular injury and hepatic inflammation, with or without fibrosis, in addition to steatosis (3). MASH increases the risk of progression to cirrhosis (14, 15) and hepatocellular carcinoma (HCC) (16), and the presence of fibrosis is the strongest predictor of disease-specific mortality (17). Thus, inflammation and fibrosis are particularly important for disease progression. These processes are driven by several nonparenchymal cell types. Liver-resident macrophages, or Kupffer cells, are activated by damage-associated molecular patterns produced by dying hepatocytes (18, 19) and other intra- and extrahepatic sources (18), and can initiate an inflammatory cascade by releasing proinflammatory factors to recruit other immune cells (20). This immune response further propagates hepatocyte damage and death and involves additional immune cell types, including monocyte-derived macrophages, neutrophils, dendritic cells, T cells, B cells, and innate lymphoid cells (21). An inflammatory environment and ongoing hepatocellular injury promote fibrosis by activating hepatic stellate cells (HSteCs). Activated HSteCs are highly proliferative, secrete abundant extracellular matrix (22), and are the primary drivers of fibrosis in MASH (23). Beyond inflammatory and fibrogenic cell types, cholangiocytes can also become activated by liver stress or injury to proliferate rapidly and secrete proinflammatory cytokines (24), which has been associated with increased MASLD severity in humans (25–27) and mouse models (28, 29). Finally, liver sinusoidal endothelial cells (LSECs) can become dysfunctional in MASLD (30–32), which may be another driver of Kupffer cell (33) and HSteC (34) activation.

Previously, we demonstrated a role for eukaryotic elongation factor 1A1 (EEF1A1) in hepatocyte lipotoxicity (35), which is an initiating event in the progression from hepatic steatosis to MASH. The canonical function of EEF1A1 is to recruit aminoacyl-tRNAs to the ribosome during peptide elongation (36). EEF1A1 also has several noncanonical functions, including regulation of the actin cytoskeleton (37–41), apoptosis (42), nuclear export (43, 44), proteolysis (45), and metabolic substrate preference (46), among others. In Western diet-induced obese mice with MASLD, we observed that inhibition of EEF1A1 with the marine compound didemnin B (DB) reduced hepatic triglyceride accumulation and steatosis (47). However, we did not determine whether decreased steatosis was associated with a change in LD size distribution. In the same study, we evaluated the effects of DB on two nonparenchymal liver cell types that contribute to inflammation and fibrosis, liver macrophages, and hepatic stellate cells. Through immunohistochemistry for F4/80 and smooth muscle α-actin, we were unable to detect significant changes in the abundance of liver macrophages and activated HSteCs in response to DB (47). However, we did observe a decrease in activated stellate cells within pancreatic islets, suggesting that DB can impact stellate cell activation within tissue (47). Furthermore, using cell culture models, we demonstrated that DB reduced the proliferation and function of monocyte- and macrophage-like cells and HSteCs (47), suggesting the potential to target these cell types with DB.

In this study, we determined the effects of EEF1A1 inhibition on hepatic LD size, bulk hepatic differential expression, and liver cell type-associated alterations in gene expression, in the same mouse tissues we used previously (47). We rigorously quantitated the effects of DB on LD size distribution and observed decreased median LD size with DB treatment. Using bulk RNA sequencing (RNA-Seq) followed by gene set enrichment analysis (GSEA) in mouse liver, we observed widespread alterations in pathways related to energy metabolism and proteostasis, among others, with DB treatment. We performed cell type deconvolution on the bulk data and identified decreased cell type association scores for cholangiocytes, mononuclear phagocytes, mesenchymal cells, plasma B cells, and endothelial cells in response to DB. In addition, through overrepresentation analysis of deconvolved bulk data using newly developed cell type marker gene sets, we further identified that hepatocytes, and to a lesser extent, cholangiocytes, were the primary contributors to differential expression in response to DB. Altogether, we have demonstrated that EEF1A1 inhibition may be effective in targeting aspects of liver biology that contribute to MASLD progression.

## METHODS

### Mouse Liver Samples Used for Analyses

Liver samples used for analyses in the present study were collected through our previous study of male 129S6/SvEvTac mice fed a Western diet (42% kcal from fat and 0.2% kcal from cholesterol) (Envigo TD.88137) for 26 wk, followed by intraperitoneal injections with DB (50 µg/kg) (Open Chemical Repository, Developmental Therapeutics Program, National Cancer Institute) (*n* = 10) or vehicle control (1% DMSO, 5.2% PEG400, 5.2% Tween 80, and 88.6% sterile saline) (*n* = 8) once every 3 days for 2 wk while being maintained on the Western diet (47). This Western diet-feeding protocol was selected because it was shown to induce obesity and elicit progression to steatohepatitis and fibrosis in 129S6 mice (48). The dose of DB was chosen because *1*) it was shown to partially inhibit protein synthesis in solid tumors in mice (49), *2*) it elicits liver concentrations that approximate the concentration of DB used in our in vitro work (80 nM), and *3*) it was sufficient to improve hepatic lipotoxicity without causing hepatotoxicity in *ob/ob* mice (50). The dosing regimen was determined based on a pharmacokinetic study of the tissue distribution of tritiated DB in mice after intraperitoneal administration showing that the half-life of DB in mouse liver is 78 h (51). Mice were euthanized by CO_2_ euthanasia between 50 and 56 h after the last treatment injection (47). All animals in each group underwent the feeding and treatment protocols simultaneously as part of a single study. This study was approved by the Animal Use Subcommittee at Western University (Protocol 2017-158) and was designed according to the Canadian Council on Animal Care guidelines .

### Hepatic Lipid Droplet Size Measurements and Size-Based Colorization

**Figure 1.**
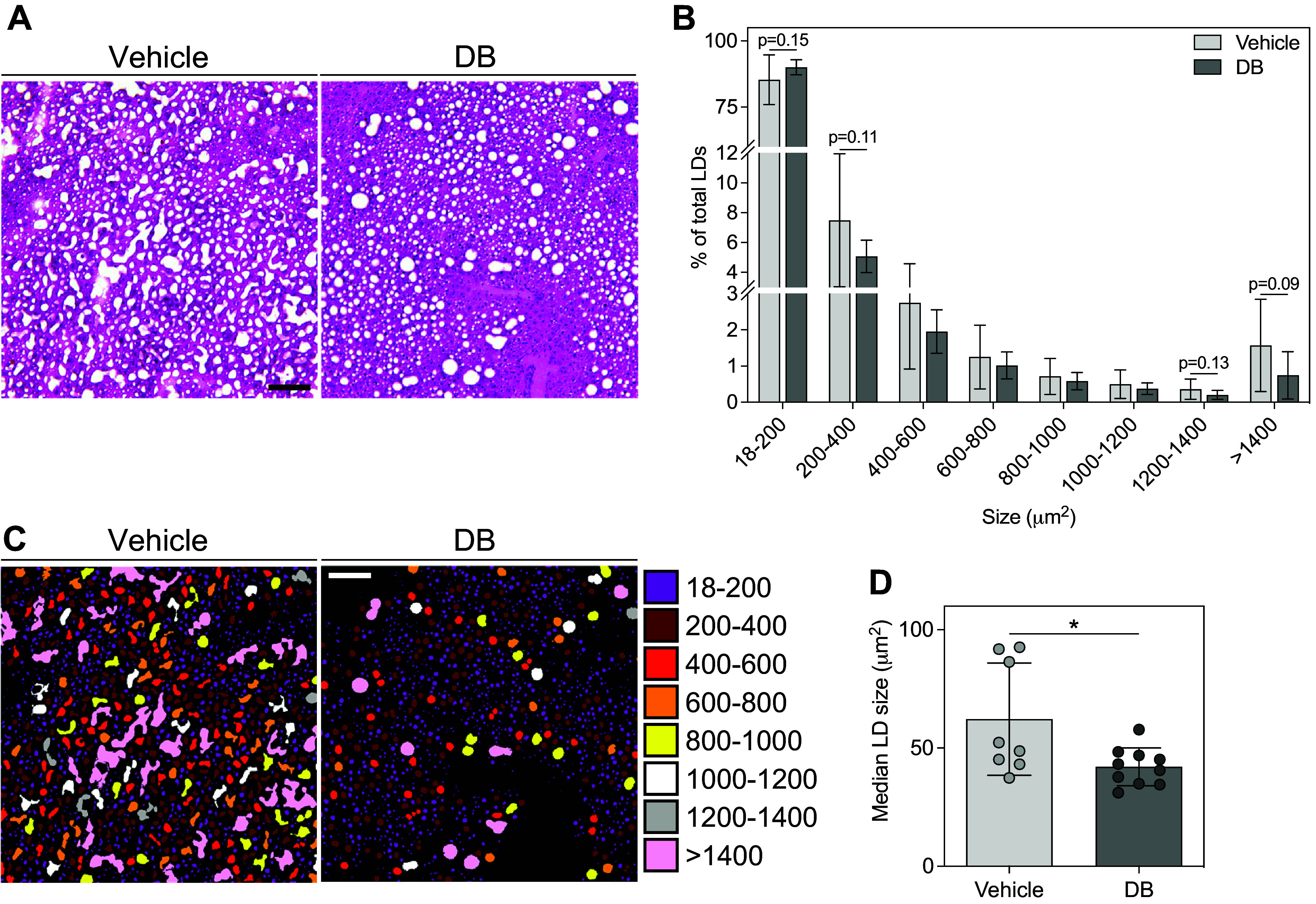
Didemnin B (DB) reduced hepatic lipid droplet size in Western diet-fed mice. *A*: light micrographs of hepatic sections from Western diet-fed mice, treated with vehicle or DB, stained with hematoxylin-eosin (H&E) to visualize steatosis and lipid droplets (LDs). *B*: LD size frequency distributions generated using ImageJ. Bars indicate means ± SD. *n* = 8–10. *C*: images in *A* colorized based on the LD size bins in *B* and on the corresponding colors in the legend. *D*: median LD size determined from data in *B*. *n* = 8–10. Bars indicate means ± SD. *P < 0.05. Scale bar = 100 µm.

LD size was determined from images captured at ×10 magnification from hematoxylin-eosin (H&E)-stained mouse hepatic sections prepared as described previously (47). Three hepatic cryosections (8 µm thickness), spaced at 40-μm intervals, were stained per mouse, and three fields of view were captured per section, resulting in nine fields of view per mouse. Image analyses were randomized and performed blind to the treatment group. An in-house developed macro (47) (Supplemental File S1; file name = “Supplemental_File_1_Liver_H&E_Fat_Droplet_Segmentation.txt”) was used to segment the white areas of the images (corresponding to LDs) from the stained areas. The macro uses the AutoThreshold plugin in ImageJ (setting: “Minimum”) to segment lipid areas from nonlipid tissue areas by automatically setting a threshold. White areas not corresponding to LDs, such as sinusoids, other vessels, or tissue sectioning artifacts, were manually excluded from images before segmentation. LDs in the segmented images were then analyzed using the particle analysis plugin in ImageJ, and LDs smaller than 18 µm^2^ (or 30 pixels) were excluded. This process was automated using a macro recorded in ImageJ (Plugins>Macros>Record), which is provided in Supplemental File S2 (file name = “Supplemental_File_2_Liver_H&E_LD_Size.ijm”). LD sizes and areas were used to generate LD size frequency distributions and calculate median LD size. Each bin in the size frequency distribution in Fig. 1*B* includes LDs with a size greater than or equal to the lower size limit and less than the upper size limit (e.g., ≥18 – <200 µm^2^, ≥200 – <400 µm^2^, etc.). Statistical comparisons using unpaired *t* tests were performed in GraphPad Prism 9.5.1. For visualization of LD sizes, representative segmented images were artificially colorized using a modification of the LD size analysis macro described previously (Supplemental File S3; file name = “Supplemental_File_3_Liver_H&E_LD_Size_With_Colourization.ijm”) that colorizes each droplet based on its corresponding size bin.

Although not used for analysis of LD size in the present study, a representative image of H&E-stained liver from a lean control mouse (fed standard chow) (47) is provided in Supplemental Fig. S1 for readers wishing to compare the appearance of steatotic mouse liver (shown in Fig. 1) to that of a normal liver.

### Bulk Liver RNA-Seq Analyses

#### RNA isolation.

RNA was isolated from snap-frozen liver tissue using the TRIzol Plus RNA Purification Kit (Thermo Fisher Scientific 12183555) following the supplier’s instructions including the optional fat removal step, with the following modifications: *1*) RNA was isolated from 35 to 50 mg of liver tissue; *2*) tissues were minced with scissors and homogenized in 500 µL of TRIzol Reagent, followed by addition of another 500 µL reagent; *3*) for fat removal, homogenates were centrifuged at 12,000 *g* at 4°C for 10 min, and 850 µL of clear infranatant was collected to a clean tube without disturbing the fat layer; *4*) after centrifugation of TRIzol/homogenate:chloroform mixture, 400–450 µL of aqueous phase was collected to a clean tube; and *5*) RNA was eluted using two sequential elutions in 75 µL RNase-free water per elution. RNA samples were stored at −80°C until use.

#### Illumina next-generation sequencing.

All samples were sequenced at the London Regional Genomics Centre (Robarts Research Institute, London, Ontario, Canada; http://www.lrgc.ca) using the Illumina NextSeq 500 (Illumina Inc.). Total RNA samples were quantified using the NanoDrop (Thermo Fisher Scientific), and quality was assessed using 1 µL (50–500 ng/µL) of sample on the Agilent 2100 Bioanalyzer (Agilent Technologies Inc.) and the RNA 6000 Nano kit (Caliper Life Sciences). They were then processed using the Vazyme VAHTS Total RNA-seq (H/M/R) Library Prep Kit for Illumina (Vazyme), which includes rRNA reduction. In brief, samples were rRNA depleted, fragmented, cDNA was synthesized, indexed, cleaned up, and amplified via PCR. Libraries were then equimolar pooled into one library and size distribution was assessed on an Agilent High Sensitivity DNA Bioanalyzer chip and quantitated using the Qubit 2.0 Fluorimeter (Thermo Fisher Scientific).

The library was sequenced on an Illumina NextSeq 500 as 76 bp single end runs, using one High Output v2 kit (75 cycles). Fastq data files were analyzed using Partek Flow. After importation, data were aligned to the *Mus musculus* genome using STAR 2.7.3a and annotated using mm10 Ensembl Transcripts release 102. Duplicated gene symbols were removed and only the copy with the highest average expression was retained. Genes with less than 10 reads across all samples were removed. Differential expression analysis was performed using the *DESeq2* package in R version 4.2.2 (52) following the corresponding vignette on Bioconductor (http://bioconductor.org/packages/devel/bioc/vignettes/DESeq2/inst/doc/DESeq2.html) [log fold change shrinkage using *apeglm* method (53); count data transformation using variance stabilizing transformation, or *vst*]. Unfiltered and filtered (fold change ≥ 1.5, adjusted *P* value <0.05) gene lists were generated. Principal component analysis was performed using the plotPCA function in *DESeq2*. A volcano plot of differentially expressed genes in DB-treated mouse liver relative to vehicle-treated mouse liver was generated using the *EnhancedVolcano* package in R (54) following the corresponding vignette on Bioconductor (https://bioconductor.org/packages/devel/bioc/vignettes/EnhancedVolcano/inst/doc/EnhancedVolcano.html).

#### Gene set enrichment analysis.

GSEA (Broad Institute, v.4.3.2) (55) was performed on *DESeq2* output following a modification of a previously described method (56). The unfiltered gene list was ranked using the *DESeq2* Wald statistic (stat column), as has been done by other groups (57). GSEA was performed using the ranked gene list and the Mouse_GOBP_AllPathways_no_GO_iea_June_03_2023_symbol.gmt gene set file (http://download.baderlab.org/EM_Genesets/). Gene sets containing greater than 200 genes and less than 15 genes were excluded, as inclusion of both large and small gene sets can complicate results interpretation (56). Gene set alterations were considered statistically significant at an adjusted *P* value <0.005. A bubble plot of significantly altered gene sets was generated using *ggplot2* in R. Similar gene sets were grouped into biological themes, and biological themes were arranged from top to bottom in the bubble plot in decreasing order based on the highest absolute normalized enrichment score within a biological theme. To display gene sets with adjusted *P* values of zero on the bubble plot, the zero-adjusted *P* values were replaced with the lowest nonzero-adjusted *P* value multiplied by 10^−1^, which is a method used by the *EnhancedVolcano* R package to handle *P* values of 0. There were many gene sets (58) significantly altered at an adjusted *P* value <0.005. To enable clear visualization of results in the bubble plot, for biological themes containing >10 gene sets (“oxidative phosphorylation, the TCA cycle, and related diseases” and “amino acid degradation and related diseases”), only the top 10 gene sets (based on highest absolute normalized enrichment score values) were displayed in the bubble plot in Fig. 3. A complete list of significantly altered gene sets can be found as separate bubble plots for upregulated and downregulated pathways in Supplemental Figs. S2 and S3, respectively.

### Deconvolution of Bulk Liver RNA-Seq to Estimate Liver Cell Types

Human cirrhotic MASLD liver single-cell RNA-Seq (scRNA-Seq) data were obtained from Ramachandran et al. (59) (https://datashare.ed.ac.uk/handle/10283/3433). To ensure appropriate mean expression per sample in the reference data could be estimated, each cell type sample combination where fewer than 10 cells were observed was removed from the data. Human genes were mapped to their mouse orthologs using Ensembl and for each many-to-one relationship, only the most highly expressed human ortholog was used. Genes detected in fewer than 20 cells were excluded. Duplicated gene symbols were removed and only the copy with the highest average expression was retained.

To reduce biases due to single-cell quality or differences in cell cycle status, mitochondrial and ribosomal transcripts and transcripts associated with G2M or S were excluded. Human G2M/S-associated transcripts were obtained from Seurat (v.3) and mapped to their mouse orthologs as described earlier.

To calculate cell type association scores, the mean expression of each gene in each reference cell type was calculated using the reference scRNA-Seq data. This was converted into a relative cell type association score by subtracting the mean expression of a given gene across all cell types. Bulk RNA-Seq data were normalized using counts per million, and expression of each cell type was scored by multiplying the normalized expression by the relative cell type association score for each cell type. Significance of differences in cell type association was determined using a two-tailed *t* test.

### KRT19 Immunohistochemistry and Image Analysis in Mouse Liver

Immunohistochemistry was performed on hepatic sections to visualize cells expressing cholangiocyte marker keratin 19 (KRT19). This work was performed using hepatic sections prepared from Western diet-fed mice treated with vehicle or DB (as described in *Mouse Liver Samples Used for Analyses*) and on hepatic sections prepared from lean control animals fed standard chow (14% kcal from fat) (Envigo 8604) and treated with vehicle (*n* = 10) for the same duration as Western diet-fed animals, as described in our previous publication (47).

Cryosections (8 µm) were prepared from optimal cutting temperature compound-embedded liver samples, mounted onto slides (Fisher Scientific 22-037-246), and stored at −80°C until use. All steps were performed at room temperature unless otherwise indicated and with calcium- and magnesium-free PBS. Immediately prior to staining, sections were thawed and air-dried for 30 min. Sections were then fixed in 4% formaldehyde in PBS for 20 min, permeabilized with 0.1% Triton X-100 in PBS for 10 min, and blocked with blocking buffer [10% goat serum (Thermo Fisher Scientific 16210072) in PBS with 0.1% Tween 20 (PBST)] for 1 h, with three PBS rinses each after fixation and permeabilization. Sections were incubated overnight at 4°C with rat monoclonal KRT19 primary antibody [Developmental Studies Hybridoma Bank (DSHB) TROMA-III-c, concentrate 0.1 mL, Lot No. 8/10/23, concentration 262 µg/mL] diluted 1/500 in blocking buffer. TROMA-III was deposited to the DSHB by Kemler R. Sections were then incubated with goat anti-rat Alexa Fluor 488-conjugated secondary antibody (Thermo Fisher Scientific A11006) diluted 1/500 in blocking buffer for 1 h. Between primary and secondary antibody incubations and after the secondary antibody incubation, sections were rinsed once with PBST, followed by 3 × 5-min washes in PBST with agitation. Sections were then counterstained with DAPI (1 µg/mL in PBST) for 5 min. Sections were rinsed once with PBST, followed by 3 × 5-min washes in PBST with agitation. This rinsing/washing step was repeated with PBS. Sections were rinsed once with deionized water, followed by 1 × 5-min wash with deionized water. Coverslips (no. 1 thickness, Fisher Scientific 12-545F) were mounted onto slides with DAKO fluorescence mounting medium (Agilent Technologies S302380-2), and slides were allowed to cure for 24 h. Images were acquired using a Leica DMI 4000B inverted microscope (objective at ×20 magnification/0.4 numerical aperture; 3.0769 pixels/µm) equipped with a Leica K5 sCMOS camera (Leica Microsystems) between 1 and 5 days after completion of staining. Acquisition parameters were as follows: resolution 2,048 × 2,048, 16-bit, green channel 1-s exposure, blue channel 150-ms exposure. Three liver sections, spaced at ≥ 40-µm intervals, were stained per mouse, and 4–9 fields of view were captured per section, resulting in 12–27 fields of view imaged and analyzed per mouse.

Images were analyzed in ImageJ by thresholding and measuring positively stained area for 488 and DAPI channel images. 488 and DAPI channel images were thresholded using the Yen and Otsu methods, respectively (Image>Adjust>Threshold>Select method as Yen or Otsu>Apply). Percent areas [(thresholded area/total image area) × 100)] for each channel were calculated using the Analyze>Measure tool. To automate the analysis, these steps were recorded using the macro recorder tool (Plugins>Macros>Record) to generate individual macros for 488 and DAPI channel images (Supplemental Files S4 and S5; 488 channel macro file name = “Supplemental_File_4_488_Area_Analysis.ijm”; DAPI channel macro file name = “Supplemental_File_5_DAPI_Area_Analysis.ijm”), which were then used to batch analyze images (Process>Batch>Macro). The 488 percent area was divided by the DAPI percent area to calculate the ratio of 488 area to DAPI area for a given image. Mean 488 area/DAPI area ratios were calculated for all images corresponding to a given mouse. Statistical comparisons using an unpaired *t* test or one-way ANOVA were performed in GraphPad Prism 10.2.3.

### Overrepresentation Analysis of Bulk Differential Expression Using Cell Type Marker Gene Sets

Cell type marker gene sets used for overrepresentation analysis were prepared from human cirrhotic MASLD scRNA-Seq data processed as described in *Deconvolution of Bulk Liver RNA-Seq to Estimate Liver Cell Types*, with the modification that the data were normalized using scran (60). Markers were identified for each cell type by comparing a gene in a given cell type to all other cell types using a Wilcoxon rank-sum test. Significance was assessed using false discovery rate (FDR) multiple testing correction. For each cell type, gene lists were filtered based on an adjusted *P* value <0.05, and then based on a fold change ≥+1.5. The filtered lists were then ordered by decreasing fold change, and the top 200 genes were selected for use in the marker gene set for a given cell type. In instances where there were fewer than 200 genes that met the filtering criteria [plasmacytoid dendritic cells (pDCs) = 197; innate lymphoid cells (ILCs) = 140; T cells = 198; mast cells = 104], all genes that did meet the criteria were used in the marker gene sets for those cell types. Marker gene sets for all 11 cell types were compiled into a single .gmt file for use in subsequent analyses (Supplemental Table S1).

Overrepresentation analysis was performed using gProfiler (v.e109_eg56_p17_1d3191d) with Bonferroni-Hochberg FDR multiple testing correction method and a significance threshold of 0.05 (61). Separate analyses were performed for upregulated [adjusted *P* value <0.05, fold change (FC) ≥ +1.5] and downregulated (adjusted *P* value <0.05, FC ≤ −1.5) differentially expressed genes to determine cell type contributions to upregulation and downregulation, respectively. An additional analysis was performed on all altered genes (adjusted *P* value < 0.05, absolute FC ≥ 1.5) to determine the primary contributors to overall differential expression irrespective of the direction of the change. Genes overlapping between the differentially expressed gene list and cell type-marker gene sets were plotted as heatmaps representing binary (black = overlap present, white = no overlap) matrices using the *ComplexHeatmap* package in R. Statistical significance is denoted by the negative log_10_-adjusted *P* values plotted in the bar plots above each heatmap.

## RESULTS

### DB Induced a Shift toward Smaller Lipid Droplets in Western Diet-Fed Mouse Liver

In our previous work, we evaluated the effects of intervention with DB in Western diet-induced obese mice with MASLD (47). The feeding protocol elicited a relatively mild disease phenotype, characterized predominantly by hepatic steatosis with minimal liver damage (indicated by plasma liver enzymes) and inflammation (indicated by histological scoring and immunohistochemistry for macrophage markers) (47). Thus, we did not observe effects of DB on markers of liver injury or inflammation in this model. We also evaluated plasma and liver lipids in our previous study, and found that DB-treated mice exhibited reduced plasma triglyceride, cholesterol, and free fatty acids, but no change in liver cholesterol (47). Importantly, treatment with DB dramatically reduced hepatic triglyceride and steatosis independently of any changes in food intake or body weight (47). Given the potential implications of LD size for disease severity (5–9) and hepatocyte function (10–12, 62), we measured LD size in H&E-stained hepatic sections from these mice (Fig. 1*A*) using an in-house developed ImageJ pipeline that performs color segmentation, particle analysis, and size-based LD colorization. Treatment with DB elicited a shift toward smaller hepatic LDs compared with vehicle (Fig. 1, *B* and *C*) and decreased median LD size (Fig. 1*D*).

### DB Altered the Expression of Genes Encoding Cytochrome P450 Metabolizing Enzymes and Stress Response Targets

**Figure 2.**
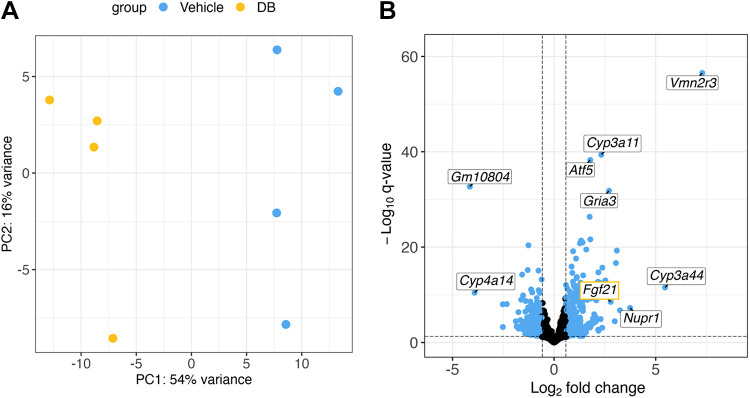
Didemnin B (DB) induced hepatic transcriptomic alterations in Western diet-fed mouse liver. *A*: principal component analysis scores plot of RNA sequencing data in the livers from Western diet-fed mice treated with vehicle (blue) and DB (yellow). *B*: volcano plot with data points representing gene transcripts altered upon treatment with DB relative to vehicle. Genes altered at an adjusted *P* value (*q* value) <0.05 and a fold change >1.5 are indicated in blue, with cutoffs denoted by dashed lines. Labels correspond to the top 5 most significantly altered genes and the genes with the top 5 largest absolute fold changes, and *Fgf21* (yellow outline), which was the tenth largest absolute fold change. *n* = 4.

To comprehensively characterize the liver transcriptome in response to DB, we used bulk RNA-Seq comparing vehicle- and DB-treated Western diet-fed mouse livers. The entire gene list containing fold changes and adjusted *P* values generated using *DESeq2* is provided (Supplemental Table S2), as is the raw data file containing read counts (Supplemental Table S3). Principal component analysis showed that most of the variance in the data is explained by treatment differences, indicating the high quality of the data (Fig. 2*A*). Genes altered in the liver in response to DB relative to vehicle were plotted based on their significance and fold change (Fig. 2*B*). Among the top five most significant and the top five largest absolute fold changes were upregulations in two genes encoding CYP3A drug metabolizing enzymes, cytochrome P450, family 3, subfamily a, polypeptide 11 (*Cyp3a11*) (fivefold) and cytochrome P450, family 3, subfamily a, polypeptide 44 (*Cyp3a44*) (44-fold). These observations are consistent with our previous finding that DB undergoes CYP3A-mediated metabolism in hepatocyte-like HepG2 cells (47). In addition, we observed upregulations in stress response genes activating transcription factor 5 (*Atf5*) (threefold), which mediates the mitochondrial unfolded protein response (63–65), and nuclear protein transcription regulator 1 (*Nupr1*) (13-fold), which is a target of the integrated stress response (66, 67), and a downregulation in cytochrome P450, family 4, subfamily a, polypeptide 14 (*Cyp4a14*) (15-fold), which catalyzes fatty acid hydroxylation (68) and has been shown to contribute to MASLD progression (58). Although not within the top five most significant or largest absolute fold changes, fibroblast growth factor 21 (*Fgf21*), which encodes a liver-derived peptide hormone known to regulate energy expenditure and lipid metabolism (69), was also highly upregulated (sevenfold) in response to DB. This finding is consistent with candidate gene expression analyses performed by real-time quantitative PCR (RT-qPCR) in our previous study (47).

### Gene Set Enrichment Analysis Identified Alterations in Pathways Related to Energy Metabolism and Proteostasis in Response to DB

**Figure 3.**
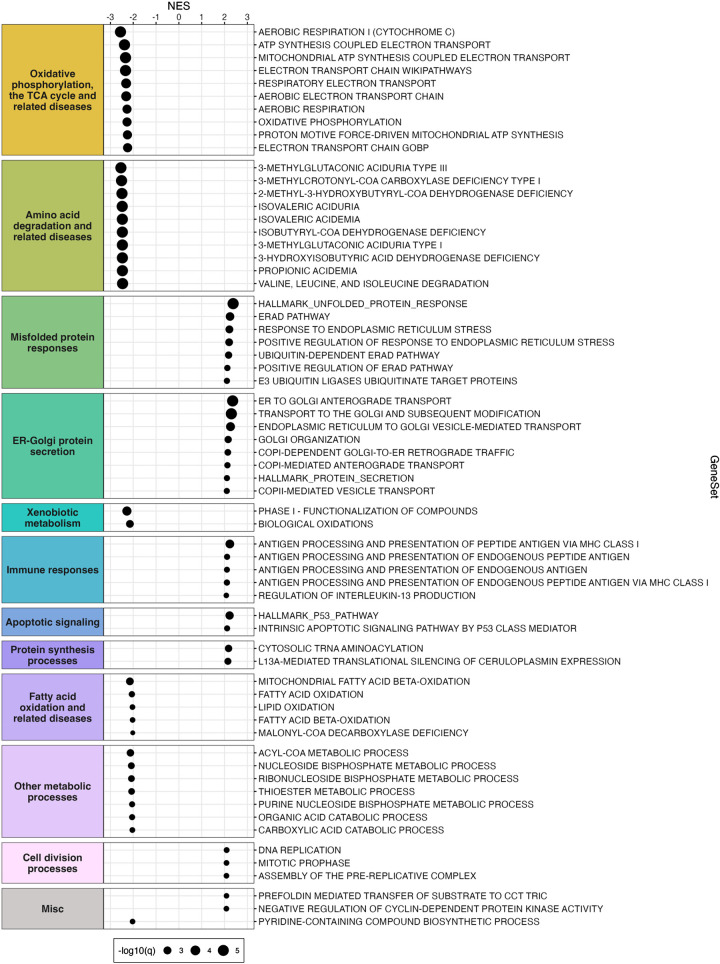
Gene set enrichment analysis identified alterations in metabolic processes, amino acid degradation, misfolded protein responses, and endoplasmic reticulum (ER)-Golgi protein secretion in response to didemnin B. Bubble plot of gene set enrichment analysis representing pathways significantly altered [adjusted *P* value (*q* value) <0.005] in the livers of didemnin B-treated mice relative to vehicle-treated mice. Normalized enrichment scores (NESs) are plotted on the *x*-axis, and gene sets are plotted on the *y*-axis. Bubble size corresponds to −log_10_(*q* value) (increases as *q* value decreases). For the biological themes “oxidative phosphorylation, the TCA cycle, and related diseases” and “amino acid degradation and related diseases,” only the top 10 gene sets (based on largest absolute NES) were included. *n* = 4.

To determine pathway alterations in response to DB, we performed GSEA, the results of which are presented as a bubble plot in Fig. 3. We observed highly significant downregulations in pathways related to metabolic processes, including oxidative phosphorylation, the TCA cycle, and fatty acid oxidation (Fig. 3; Supplemental Fig. S3); this is likely explained by the fact that DB-treated mouse livers contain substantially less lipid (47) and therefore less metabolic substrate for these pathways. In addition, we observed alterations in pathways related to proteostasis. Pathways related to amino acid degradation were downregulated, and pathways related to misfolded protein responses, endoplasmic reticulum (ER)-Golgi protein secretion, and protein synthesis were upregulated (Fig. 3; Supplemental Figs. S2 and S3). These findings suggest that DB perturbed proteostasis and/or induced an ER stress response, which is consistent with gene expression analyses performed by RT-qPCR in our previous study (47).

### DB Decreased Cell Type Association Scores for Several Cell Types, Including Cholangiocytes

**Figure 4.**
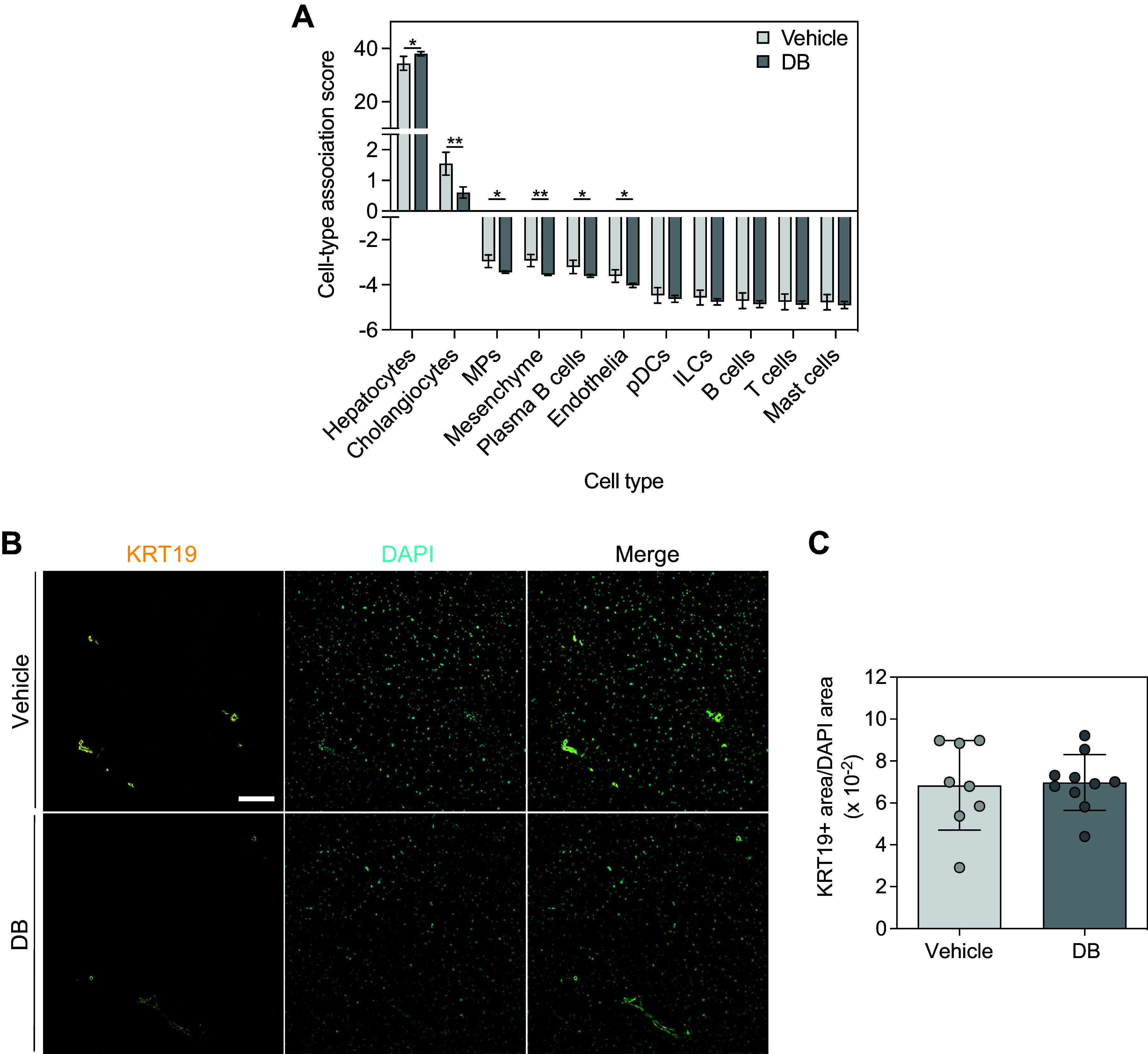
Didemnin B (DB) increased the cell type association score for hepatocytes and decreased cell type association scores for cholangiocytes, mononuclear phagocytes, mesenchymal cells, plasma B cells, and endothelial cells in mouse liver. *A*: relative cell type association scores were calculated from reference single-cell RNA-Seq data from human cirrhotic metabolic dysfunction-associated steatotic liver disease (MASLD) liver where genes have been mapped to mouse orthologs. Bulk RNA-Seq data from vehicle- and DB-treated Western diet-fed mouse liver were normalized using counts per million, and expression of each cell type was scored by multiplying the normalized expression by the relative cell type association score for each cell type. *n* = 4. Data are means ± SD. *B*: fluorescent micrographs of hepatic sections from Western diet-fed mice, treated with vehicle or DB, stained for KRT19 (yellow), and counterstained with DAPI (cyan). Scale bar = 100 μm. *C*: KRT19+ area normalized to DAPI area analyzed in ImageJ. *n* = 8–10. Bars indicate means ± SD. *P < 0.05; **P < 0.01.

Considering our prior findings that DB reduced proliferation of monocytes and activated HSteCs in vitro (47), we were interested in gaining a broader perspective of potential changes in liver cell type composition in response to DB. Using publicly available scRNA-Seq data from human cirrhotic MASLD liver (59) (where human genes were mapped to their mouse orthologs) as the reference data, cell type association scores were calculated from the normalized expression values in our bulk RNA-Seq data. We observed a significant increase in the hepatocyte cell type association score and significant decreases in cell type association scores for cholangiocytes, mononuclear phagocytes [blood or tissue monocytes, Kupffer cells, conventional dendritic cells, scar-associated macrophages (59)], mesenchymal cells [HSteCs, scar-associated mesenchymal cells, vascular smooth muscle cells, and mesothelial cells (59)], plasma B cells, and endothelial cells in response to DB (Fig. 4*A*).

In light of the known expansion of cholangiocytes that occurs in MASLD in humans and mouse models (25–29) and the approximately threefold reduction in the cholangiocyte cell type association score elicited by DB treatment (Fig. 4*A*), we further investigated this finding by immunohistochemistry for cholangiocyte marker KRT19 in cryosections prepared from Western diet-fed vehicle- and DB-treated mouse livers. We also included cryosections prepared from livers of standard chow-fed vehicle-treated mice to evaluate whether expansion of cholangiocytes occurred in response to Western diet feeding, as has been shown in other studies. We observed increased liver KRT19-positive area in Western diet-fed mice compared with standard chow-fed mice (Supplemental Fig. 4, *A* and *B*). However, we did not detect alterations in KRT19-positive area in response to DB (Fig. 4, *B* and *C*). Therefore, although DB reduced the cholangiocyte-associated gene expression signature, we were not able to confirm this by measuring KRT19-positive cells. This is consistent with a lack of differential expression of *Krt19* in response to DB in the bulk RNA-Seq data (fold change = −1.04; adjusted *P* value = 0.41) (Supplemental Table S2). The discrepancy between the cell type deconvolution finding and KRT19 abundance may relate to the limitations of using KRT19 as a marker for cholangiocytes.

### Hepatocyte and Cholangiocyte Marker Genes Were Overrepresented among Genes Differentially Expressed in Response to DB

**Figure 5.**
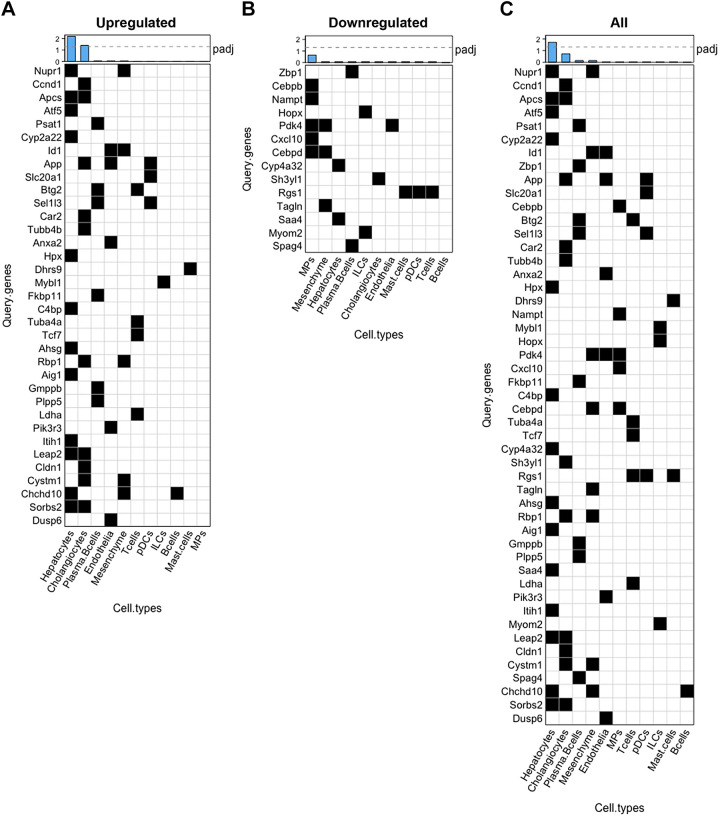
Marker genes for hepatocytes and cholangiocytes were overrepresented among genes differentially expressed in response to didemnin B (DB). Cell type marker gene sets were generated from reference scRNA-Seq data by selecting the top 200 most upregulated genes [fold change (FC) ≥1.5, adjusted *P* value <0.05] in a given cell type. Overrepresentation analysis was performed in gProfiler using genes significantly upregulated (FC ≥ +1.5, adjusted *P* value <0.05) (*A*), genes significantly downregulated (FC ≤ −1.5, adjusted *P* value <0.05) (*B*), and all significantly altered genes (absolute FC ≥ 1.5, adjusted *P* value < 0.05) in mouse liver in response to DB as query genes (*C*). Results are presented as heatmaps that represent binary matrices, indicating the presence or absence of query genes within a cell type marker gene set (black = present, white = absent). Negative log_10_-adjusted *P* values are displayed as bar plots above each heatmap, and the dashed line represents an adjusted *P* value <0.05. Significantly overrepresented cell type marker gene sets are indicated by negative log_10_-adjusted *P* values above this line. *n* = 4.

Given the changes in estimates of liver cell type proportions we observed, we next determined which cell types predominantly contributed to the differential expression induced by DB. Hepatocytes constitute 70%–80% of total liver mass (70), thus it seemed likely that most changes detected by bulk RNA-Seq would be driven by hepatocytes. However, changes in cell type composition could, in part, drive differential expression as well. Using scRNA-Seq data from human cirrhotic MASLD liver (59) , we generated cell type marker gene sets for all 11 liver cell types identified in that study. We then evaluated whether these marker gene sets were overrepresented among genes differentially expressed in mouse liver in response to DB. Results from the overrepresentation analyses are presented as heatmaps that represent binary matrices indicating the presence or absence of query genes within a cell type marker gene set (Fig. 5). Heatmaps for upregulated (adjusted *P* value <0.05; FC ≥ +1.5) (Fig. 5*A*), downregulated (adjusted *P* value <0.05; FC ≤ −1.5) (Fig. 5*B*), and all altered (adjusted *P* value < 0.05; absolute FC ≥ 1.5) (Fig. 5*C*) differ in length because, of the 290 upregulated genes and 243 downregulated genes, only 35 and 14 genes, respectively, overlapped with genes in the cell type-marker gene sets. Hepatocytes and cholangiocytes were significantly overrepresented among the upregulated genes, with 12 and 10 genes overlapping with differentially expressed genes, respectively (Fig. 5*A*). No cell types were significantly overrepresented among the downregulated genes, but mononuclear phagocytes overlapped the most with differentially expressed genes (5 genes of the 14 query genes were part of the mononuclear phagocyte gene set) (Fig. 5*B*). Among all altered genes, only hepatocytes were significantly overrepresented, with 14 genes overlapping with query genes (Fig. 5*C*). Altogether, these findings suggest that hepatocytes and cholangiocytes are the primary drivers of upregulated gene expression in the bulk data, and that hepatocytes predominantly drive overall gene expression changes.

## DISCUSSION

We previously demonstrated that inhibition of EEF1A1 with DB reduced hepatic steatosis in diet-induced obese mice with MASLD, and furthermore that DB decreased the proliferation and function in vitro of cell types that would contribute to liver inflammation and fibrosis (47). However, questions remained as to the effects of EEF1A1 inhibition on LD size and liver cell type-associated gene expression changes, which have implications for MASLD progression. We measured LD size in H&E-stained liver tissue sections using an automated ImageJ pipeline and observed a clear shift toward smaller LDs in response to DB. We performed bulk RNA-Seq followed by GSEA, which revealed widespread alterations in pathways related to energy metabolism and proteostasis with DB treatment. Using publicly available scRNA-Seq data as a reference, we deconvolved the bulk RNA-Seq data to estimate liver cell type proportions and observed decreased scores for cholangiocytes, mononuclear phagocytes, mesenchymal cells, plasma B cells, and endothelial cells. We further used these publicly available scRNA-Seq data to generate cell type marker gene sets for use in overrepresentation analysis. This new approach revealed that hepatocytes, and to a lesser extent, cholangiocytes, are the main contributors to bulk differential expression in mouse liver in response to DB. Beyond identifying potentially beneficial effects of DB on LD size and liver cell type-associated gene expression, our work is among the first to use bulk RNA-Seq deconvolution to estimate changes in liver cell type proportions in response to a pharmacological treatment. Furthermore, we demonstrate the utility of this approach to gain further information about cell type changes in preclinical studies where bulk RNA-Seq, but not scRNA-Seq, has been performed.

Our finding that DB promoted a shift toward smaller hepatic LDs may have important implications with respect to disease progression and hepatocyte function. In patients with MASLD, the presence of microsteatosis has been associated with more advanced histological disease (5). Although we did demonstrate smaller LDs in this study, they did not seem to impart a foamy appearance to hepatocytes (Fig. 1*A*), suggesting that this may not constitute true microsteatosis (4). In fact, the tissue architecture appears to be less disrupted by steatosis in DB-treated livers compared with those treated with vehicle (Fig. 1*A*). Given the detrimental effects of large LDs on cellular processes (6, 8, 10–12), it seems reasonable that the shift toward smaller LDs in response to DB in this mouse model may provide benefit with respect to hepatocyte function. This conclusion is consistent with our previous observations of decreased plasma triglycerides, decreased plasma insulin, and improved glucose tolerance with DB treatment, suggesting improved liver metabolic function (47).

Based on our bulk RNA-Seq analyses, we can speculate as to potential mechanisms through which inhibition of EEF1A1 with DB reduced hepatic steatosis. We observed that DB upregulated the stress response genes, *Nupr1* and *Atf5*. We also observed marked upregulation of *Fgf21*, which encodes a peptide hormone known to regulate lipid metabolism and energy expenditure (69) and is a target of the integrated stress response (71). In addition, GSEA revealed upregulation of pathways related to misfolded protein responses [unfolded protein response, ER stress, and ER-associated degradation], ER-Golgi protein secretion, cytosolic tRNA aminoacylation, and downregulation of pathways related to amino acid degradation, in response to DB. Taken together, these observations may suggest that DB induced a stress response related to amino acid or protein restriction, which would be consistent with our previous observation of decreased total hepatic protein with DB treatment (47). Interestingly, protein restriction has been shown to increase energy expenditure and reduce fat mass by promoting production of liver-derived FGF21 (69, 72–74). This is thought to occur through an integrated stress response that requires the stress sensor general control nonderepressible 2 (GCN2) (72, 73) or NUPR1 (74). Moreover, induction of FGF21 in response to protein restriction appears to be required for the associated reduction in hepatic steatosis (75–77). Based on this combined knowledge, we suggest that by causing hepatic protein restriction, DB may have initiated a stress response resulting in FGF21 production by the liver, ultimately reducing hepatic steatosis.

We also deconvolved our bulk liver RNA-Seq data using a method developed in-house to estimate proportions of liver cell types. Other groups have deconvolved bulk RNA-Seq data to characterize cell type changes in human (78–81) and mouse (82) liver disease, and to identify cell type-associated regulatory variants in human liver using publicly available data (83). However, to our knowledge, only two other studies have used bulk RNA-Seq deconvolution to demonstrate changes in hepatic cellular composition in response to a pharmacological intervention (84, 85). This new application of RNA-Seq deconvolution could be routinely used to glean further information about tissue cell type composition from pre-existing bulk RNA-Seq data or in experiments where scRNA-Seq is not feasible.

Our deconvolution method revealed that DB decreased cell type association scores for mononuclear phagocytes, mesenchymal cells, cholangiocytes, plasma B cells, and endothelial cells. The decrease in scores for mononuclear phagocytes and mesenchymal cells is consistent with our previous work indicating the ability of DB to target monocytes/macrophages and HSteCs in vitro (47). Given the important contributions of immune cells, mesenchymal cells, and cholangiocytes to MASLD progression (20–34, 86–91), it seems reasonable that decreased abundance of these cell types in response to DB would be beneficial.

Of the cell type association scores altered by treatment with DB, the score for cholangiocytes was the most dramatically affected (Fig. 4*A*). Thus, we further investigated this finding by performing immunohistochemistry for cholangiocyte marker KRT19 in mouse liver sections. Our finding of no effect of DB on KRT19-positive staining (Fig. 4*C*) may result from limitations of using KRT19 as a marker for cholangiocytes. Although KRT19 is extensively used as a biliary marker, bile ducts have variable expression of KRT19 in rats (92) and in humans (93). Furthermore, recent scRNA-Seq studies in mouse liver have shown that only certain cholangiocyte populations have high expression of *Krt19* (94, 95). Therefore, our immunohistochemical analyses excluded the detection of potential effects on KRT19-negative cholangiocytes. Other cholangiocyte markers, such as keratin 7 (KRT7) or epithelial cell adhesion molecule (EPCAM), could be considered; although these markers are also not uniformly expressed across all cholangiocytes (96). Accurate and comprehensive measurement of cholangiocytes in tissue would require the use of a number of cholangiocyte markers to overcome the heterogeneity in the population. In this respect, the deconvolution approach we used in this study is advantageous, because it measures the association between our bulk data and a near-complete cholangiocyte gene expression profile. However, in future work, cell type deconvolution findings could be validated using immunohistochemistry for an array of cholangiocyte markers to maximize the proportion of cholangiocytes measured. Based on our previous finding that DB dramatically slows the proliferation of cell types with low drug-metabolizing capacity, but not hepatocyte-like cells with high drug-metabolizing capacity (47), it seems likely that DB would have similar antiproliferative effects on other nonparenchymal cells within the liver, such as cholangiocytes. Considering the known contribution of cholangiocyte activation to MASLD severity (38, 43), targeting this cell type could be an important aspect of the potential therapeutic effect of DB.

An important limitation of the cell type deconvolution performed in the present study was the use of scRNA-Seq data generated from human cirrhotic MASLD liver as the reference data set. Although we mapped human genes to their mouse orthologs prior to using the scRNA-Seq data for deconvolution, this would not account for differences in gene expression profiles for a given cell type between mice and humans. For example, ANXA4 is selectively enriched and uniformly expressed in human cholangiocytes, but is not specific and has variable expression in mouse cholangiocytes (https://www.livercellatlas.org). In addition to species differences, the reference data set was derived from livers with MASLD-related cirrhosis (59), a disease stage distinct from the early-stage, prefibrotic MASLD in the mice used in the present study (47). Ramachandran et al. (59) demonstrated expansion of several hepatic subpopulations in human cirrhosis, which likely differs from the cell type composition observed at earlier disease stages. We used these data as the reference as this was the highest quality, most comprehensive data set available, but future work will use mouse scRNA-Seq datasets that better mimic the disease severity of our mouse model as they become available.

Our finding that hepatocytes were overrepresented among genes differentially expressed in response to DB in mouse liver is not surprising, given that hepatocytes constitute 70%–80% of liver volume (70). This analysis does, however, allow us to speculate about cell type contributions to the potential mechanisms described earlier. For example, *Atf5*, a stress response gene, was highly significantly upregulated in response to DB (Fig. 2*B*) and is exclusively present in the hepatocyte marker gene set (Fig. 5*A*), suggesting that this upregulation was most likely driven by hepatocytes. In contrast, *Nupr1*, another stress response gene significantly upregulated in response to DB (Fig. 2*B*), is present both in hepatocyte and mesenchymal cell marker gene sets (Fig. 5*A*); therefore, this induction could have been driven by either or both cell types. The finding that cholangiocytes are overrepresented among the upregulated genes (Fig. 5*A*) may seem paradoxical considering the threefold decrease in the cholangiocyte cell type association score in response to DB (Fig. 4). However, DB could both reduce total cholangiocyte numbers and strongly upregulate gene expression in the cholangiocytes that are still present. In fact, both findings suggest that DB may specifically accumulate in and target these cells, compared with other nonparenchymal cell types.

### Conclusions

We determined that EEF1A1 inhibition with DB decreased hepatic LD size and that associated changes in hepatic gene expression were primarily attributable to hepatocytes and cholangiocytes. We further revealed possible stress response-related mechanisms through which DB may decrease hepatic steatosis and supported the potential for DB to target nonparenchymal cell types, particularly cholangiocytes, that contribute to MASLD progression. This work highlights the therapeutic potential of targeting EEF1A1 in the setting of MASLD and the utility of RNA-Seq deconvolution to reveal valuable information about tissue cell type composition and cell type-associated gene expression from bulk RNA-Seq data.

## DATA AVAILABILITY

All data generated in this study are included in this article and associated supplemental material.

## SUPPLEMENTAL MATERIAL

10.6084/m9.figshare.26307979 Supplemental Figs. S1–S4: https://doi.org/10.6084/m9.figshare.26307979.

10.6084/m9.figshare.26311759 Supplemental Table S1: https://doi.org/10.6084/m9.figshare.26311759.

10.6084/m9.figshare.26311924 Supplemental Table S2: https://doi.org/10.6084/m9.figshare.26311924.

10.6084/m9.figshare.26311939 Supplemental Table S3: https://doi.org/10.6084/m9.figshare.26311939.

10.6084/m9.figshare.26311951 Supplemental Files S1–S5: https://doi.org/10.6084/m9.figshare.26311951.

## GRANTS

This work was supported by the Natural Sciences and Engineering Research Council of Canada Grants RGPIN-2017-04646 and RGPIN-2023-03550 (to N.M.B.) and the Canadian Institutes of Health Research Grants 149010 (to N.M.B.) and 152944 (to R.W.). R.B.W. was supported by a Natural Sciences and Engineering Research Council of Canada Doctoral Scholarship. Y.J.C. was supported by a Dean’s Undergraduate Research Opportunity Program summer research award.

## DISCLOSURES

No conflicts of interest, financial or otherwise, are declared by the authors.

## AUTHOR CONTRIBUTIONS

R.B.W., R.W., and N.M.B. conceived and designed research; R.B.W. performed experiments; R.B.W., Y.J.C., R.Z., S.M., and T.S.A. analyzed data; R.B.W., T.S.A., R.W., and N.M.B. interpreted results of experiments; R.B.W. and T.S.A. prepared figures; R.B.W. drafted manuscript; R.B.W., T.S.A., R.W., and N.M.B. edited and revised manuscript; R.B.W., Y.J.C., R.Z., S.M., T.S.A., R.W., and N.M.B. approved final version of manuscript.
